# Organ-specific and stage-dependent aboveground stoichiometric economics in *Fargesia denudata*

**DOI:** 10.3389/fpls.2026.1882972

**Published:** 2026-07-21

**Authors:** Chenhui Qu, Siyu Liu, Yanhong Liu

**Affiliations:** 1 School of Ecology and Nature Conservation, Beijing Forestry University, Beijing, China; 2 Hebei Normal University, Shijiazhuang, China

**Keywords:** bamboo growth, clonal integration, ontogeny, organ modularity, staple bamboo, stoichiometric trait

## Abstract

**Introduction:**

Plant stoichiometric traits can indicate nutrient allocation and resource-use strategies, but their interpretation depends on organ identity and developmental stage. *Fargesia denudata* is a staple bamboo for giant pandas and an important clonal understory plant in subalpine forests. We examined how C, N, and P concentrations and their ratios are coordinated across aboveground organs and ontogeny, and how stage-specific stoichiometric economics is associated with environmental and community variables.

**Methods:**

Across 52 plots in Wanglang National Nature Reserve, including 37 plots with *F. denudata*, we measured stoichiometric traits in shoot culms and in mature culms, leaves, and twigs, together with topographic, soil, and community variables.

**Results:**

Stoichiometric coordination was modular and stage-specific, with limited independent within-organ coordination retained only in shoot culms and mature twigs, and cross-organ links suggesting that mature twigs acted as the main bridge between culms and leaves. PC1 axes generally separated N- and P-enriched stoichiometry from ratio-enriched stoichiometry, indicating comparable but developmentally reorganized stoichiometric-economic axes. Shoot culms occupied the nutrient-enriched, relatively acquisitive end of the axis, whereas mature culms shifted toward the ratio-enriched, more conservative end. Shoot-culm PC1 did not scale significantly into mature organ-level or integrated mature aboveground PC1, indicating developmental reorganization rather than direct continuity. In mature bamboo, aboveground stoichiometric economics was assembled through organ-specific contributions, with twigs forming the main link between culms and leaves. Plots with *F. denudata* occurred on steeper slopes and had higher soil C concentration than plots without *F. denudata*. Environmental and community associations were stage-dependent and were concentrated mainly in shoot culms. Elevation showed a significant positive total effect on shoot-culm PC1, corresponding to a shift toward a more conservative stoichiometric strategy along the elevational gradient. Mature aboveground PC1 showed a more limited pattern, with only a single correlation with soil pH.

**Discussion:**

These results suggest that stoichiometric strategies in *F. denudata* are shaped by organ-specific coordination, ontogenetic reorganization, and stage-dependent links with environmental and community conditions. Organ- and stage-specific stoichiometric indicators may inform bamboo resource assessment and giant panda habitat evaluation.

## 1 Introduction

Economic spectra emerge when functional traits covary in ways that reflect coordinated resource investment (Wright et al., 2004; Reich and Cornelissen, 2014). For stoichiometric traits, such coordination can generate a dominant nutrient-driven axis that contrasts a nutrient-enriched strategy characterized by higher N and P, and lower C:N ratio with a more conservative strategy (Elser et al., 2010). The stoichiometric-economic spectrum may extend beyond single organs to the aboveground system as a whole (Li et al., 2022). However, such extension need not imply uniform integration across organs and developmental stages. Integration may weaken across ontogeny if trait means and covariances shift during development (Spasojevic et al., 2014; Henn and Damschen, 2021), or across organs if differentiation limits coordination and yields modular structure even when an integrated axis is detectable (Armbruster et al., 2014; Li et al., 2022). This issue is closely related to compartmentalized homeostasis, in which elemental regulation is decoupled among tissues and elements. Recent work on Moso bamboo has proposed that this strategy can maintain strict leaf N:P homeostasis while permitting greater P flexibility in woody tissues under nutrient imbalance (Wang et al., 2026). Thus, an aboveground stoichiometric economics axis, if present, may reflect coordinated but uneven contributions of different organs rather than uniform stoichiometric responses across tissues. In addition, the environmental and community correlates of stoichiometric economics may differ between developmental stages (Funk et al., 2020; Barton, 2024). The key question is therefore whether stoichiometric economics remains integrated across organs and ontogeny, or is reassembled as organ composition changes.

This question is difficult to address in most plants because a single aboveground plant rarely expresses different states at the same time (Barton, 2024). As a result, developmental strategies are usually inferred from comparisons among separate plants (Hu et al., 2023; Liu et al., 2025). Clonal plants provide a notable exception. Through a shared rhizome system, shoot and mature ramets can coexist within a single clonal genet while sharing much of the same genetic background (Liese and Köhl, 2015). This makes it possible to examine ontogenetic reassembly of stoichiometric economics within a shared clonal system rather than across separate plants.

Bamboos are especially informative because ontogenetic change is accompanied by a marked shift in aboveground organ composition (Liese and Köhl, 2015). During the shoot stage, aboveground structure and investment are largely culm-driven. In mature bamboo ramets, aboveground function depends on the coordinated contributions of culms, leaves, and twigs (Yen, 2016; Su et al., 2026). This shift makes it unclear whether stoichiometric economics in shoot culms scales directly to mature aboveground stoichiometric economics or instead reflects a distinct stage of aboveground stoichiometric economics assembly. It also suggests that the environmental and community sensitivity of stoichiometric economics may differ between shoot and mature stages.

*Fargesia denudata* is a clonal understory bamboo and one of the major staple bamboo species of the giant panda. Existing work on *F. denudata* has focused mainly on size- and biomass-related variation (Wu et al., 2005; Li et al., 2013; Kang et al., 2019; Xiong et al., 2022; Li et al., 2023a; Zhao et al., 2025), traits related to panda feeding (Wang et al., 2017; Zhang et al., 2020; Shi et al., 2025; Chen et al., 2026), and some studies using omics-based approaches (Zhang et al., 2017; Xiang et al., 2024). By contrast, systematic work on its stoichiometric traits and economics remains limited. *F. denudata* therefore provides a useful system for testing whether aboveground stoichiometric economics is integrated across organs, reorganized across ontogeny, and differentially associated with environmental and community variables across stages.

Here, we asked three linked questions. First, how are aboveground stoichiometric traits coordinated within and among organs, and do these coordination patterns form a common stoichiometric-economic spectrum or organ-specific modules? Second, does shoot-culm stoichiometric economics scale into mature organ-level and integrated mature aboveground stoichiometric economics, or is the mature aboveground spectrum assembled through organ-specific contributions? Third, do environmental and community associations with stoichiometric economics differ between shoot and mature stages, and which topographic, soil, and community variables are associated with stage-specific economic strategies? By treating stoichiometric economics as an organ- and stage-specific assembly problem, this study aims to clarify how stoichiometric strategies are coordinated, reorganized across ontogeny, and linked to environmental and community conditions in *F. denudata*, a staple bamboo for giant pandas.

## 2 Materials and methods

### 2.1 Study area

**Figure 1:**
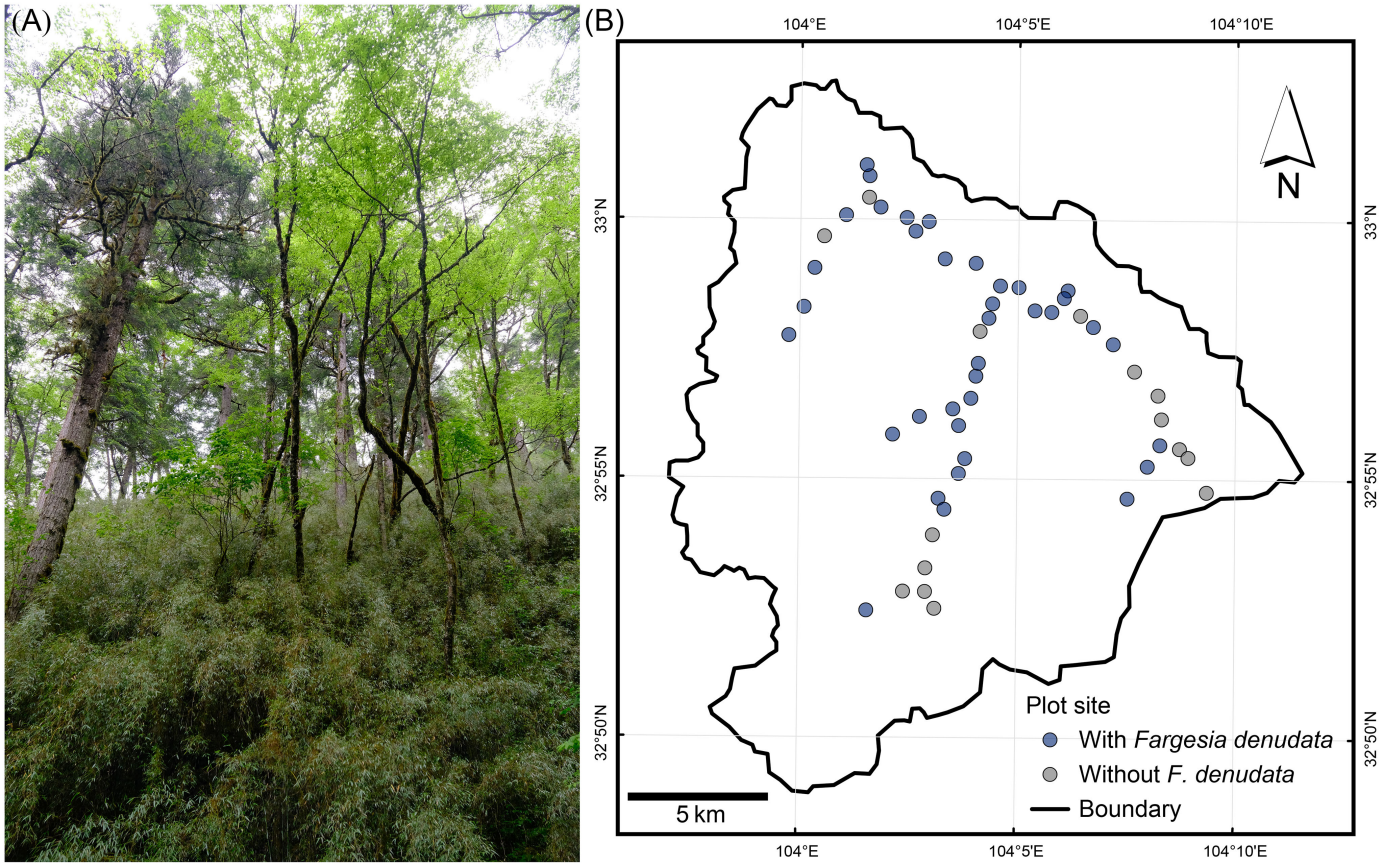
**(A)** Representative habitat of *Fargesia denudata* and **(B)** distribution of sampling plots in Wanglang National Nature Reserve.

Wanglang National Nature Reserve (103°57′0.94″–104°11′25.9″E, 32°48′56″–33°2′38.9″N) is located in Pingwu County, Sichuan Province, China. It covers 322.97 km^2^ and spans an elevation range of 2300–4983 m. The reserve is an important habitat for the giant panda, with 28 individuals recorded in the area (National Forestry and Grassland Administration, 2021), and its staple bamboo is primarily *Fargesia denudata*. *F. denudata* here occurs in forest understories at elevations of 2300–3500 m (**Figure 1A**), growing mainly in mountain brown soils and mountain dark brown soils (Li, 2018).

### 2.2 Plot establishment and field sampling

From July to September 2024, 52 sampling plots were established within the reserve, including 37 plots with *F. denudata* and 15 plots without *F. denudata*, with a minimum distance of 300 m between plots (**Figure 1B**). Each plot was 20 m × 20 m and was used for tree-layer surveys. Within each plot, 5 m × 5 m shrub subplots and soil sampling points were arranged using a five-point sampling design (Fang et al., 2009). Environmental information, including longitude, latitude, elevation, aspect, slope, and soil type, was recorded for each plot.

*F. denudata* ramets were classified into shoot and mature developmental stages. For each stage, the available aboveground organs were sampled. Shoot-stage ramets were defined as unbranched bamboo shoots before twig and leaf emergence, with the culm as the only aboveground organ. Therefore, only shoot-culm samples were collected from shoot-stage ramets. Mature ramets were defined as green-culmed ramets with well-developed twigs and fully expanded leaves. For mature ramets, culm, leaf, and twig samples were collected. In plots with *F. denudata*, 3–10 ramets per available developmental stage were sampled, depending on local abundance and ramet availability.

The tree layer was defined as woody plants with a diameter at breast height (DBH) greater than 10 cm. DBH and height were measured for every tree individual. The shrub layer was defined as woody plants with DBH < 10 cm and height > 1 m. Basal diameter and height were measured for every shrub individual. Species names of tree and shrub individuals were also recorded.

Topsoil (0–20 cm) at each plot was collected from five sampling points, after removing surface litter. The five subsamples were thoroughly mixed to form one composite soil sample per plot. All samples were stored in sealed bags at 4 °C and transported to the laboratory for further analysis.

### 2.3 Trait and soil variable measurements

A total of 18 stoichiometric traits associated with stoichiometric economic strategies were measured (**Table 1**). For most traits, measurements followed standardized protocols (Pérez-Harguindeguy et al., 2013). To determine total C, total N, and total P concentrations in aboveground organs of bamboo and the leaves of other community species, samples were oven-dried at 65 °C to constant mass and finely ground using a ball mill (AM100S, Ants Scientific Instruments). Total C and N concentrations were quantified using an elemental analyzer (VARIO EL III, Elementar), and total P concentrations were determined using a continuous flow analyzer (San++, Skalar) after H_2_SO_4_–H_2_O_2_ digestion.

**Table 1:** List of 18 stoichiometric traits measured in this study.

Organ	Trait	Unit
Culm	Culm C concentration (Culm C)	g/kg
	Culm N concentration (Culm N)	mg/g
	Culm P concentration (Culm P)	mg/g
	Culm C:N ratio (Culm CN)	–
	Culm C:P ratio (Culm CP)	–
	Culm N:P ratio (Culm NP)	–
Leaf	Leaf C concentration (Leaf C)	g/kg
	Leaf N concentration (Leaf N)	mg/g
	Leaf P concentration (Leaf P)	mg/g
	Leaf C:N ratio (Leaf CN)	–
	Leaf C:P ratio (Leaf CP)	–
	Leaf N:P ratio (Leaf NP)	–
Twig	Twig C concentration (Twig C)	g/kg
	Twig N concentration (Twig N)	mg/g
	Twig P concentration (Twig P)	mg/g
	Twig C:N ratio (Twig CN)	–
	Twig C:P ratio (Twig CP)	–
	Twig N:P ratio (Twig NP)	–

For the measurement of soil physicochemical properties, air-dried soil samples were crushed and passed through a 2 mm sieve to remove roots and stones. Soil pH and electrical conductivity (Soil EC) were determined using the <2-mm soil fraction at soil-to-water ratios of 1:2.5 and 1:5, respectively, with a dual-parameter meter (P904, Yoke Instrument). The <2-mm soil fraction was finely ground and passed through a 0.150 mm sieve for the determination of soil total C (Soil C), total N (Soil N), and total P (Soil P). Soil C and Soil N were quantified using an elemental analyzer (VARIO EL III, Elementar), and Soil P was determined using a continuous flow analyzer (San++, Skalar) after H_2_SO_4_–H_2_O_2_ digestion.

### 2.4 Statistical analyses

Before analysis, ramet-level trait measurements were averaged within each plot to obtain plot-mean trait values. All trait-based analyses were conducted at the plot level to match the scale of environmental and community variables and to avoid pseudoreplication.

To characterize relationships among stoichiometric traits within and across organs, we calculated Spearman’s rank correlation coefficients and constructed a multilayer network using the R package PTMN (He et al., 2026). To avoid spurious correlations caused by mathematical coupling, non-independent within-organ stoichiometric trait pairs were excluded before network construction, including traits directly used to calculate ratios and ratios sharing common components. Cross-organ trait pairs were retained because they were derived from organ-specific measurements and were therefore mathematically independent. To account for multiple testing, raw *p* values were adjusted using the Benjamini–Hochberg false discovery rate (FDR) procedure. We focused on significant edges with FDR-adjusted *p* ≤ 0.05 and |r| > 0.2.

To quantify covariation among stoichiometric traits and assess whether a stoichiometric economics spectrum was expressed at organ and aboveground levels in *F. denudata*, we performed principal component analysis (PCA) using the R package FactoMineR based on z-score standardized trait variables (Lê et al., 2008). We focused on PC1, which captured the largest proportion of variance and represented the dominant stoichiometric-economic axis. When the PC1 axis of a specific organ was oriented oppositely to that of the mature aboveground system, the signs of its PCA scores and loadings were reversed (multiplied by -1). To compare culm economics (PC1) between shoot and mature culms, we used paired Student’s t-tests or Wilcoxon signed-rank tests, depending on whether the paired differences met the assumption of normality (Li, 2000).

To test scaling relationships of PC1 scores at organ and aboveground levels, we fitted ordinary least squares linear regressions (Li, 2000). Specifically, we regressed (i) shoot-culm PC1 against mature culm, leaf, twig, and mature aboveground PC1 scores; (ii) mature culm PC1 against mature twig and leaf PC1 scores; and (iii) mature aboveground PC1 against organ-level PC1 scores.

Differences in environmental and community variables between plots with and without *F. denudata* were tested using Student’s t-tests or Mann–Whitney U tests, depending on assumptions of normality and homoscedasticity (Li, 2000).

To evaluate potential predictors of stage-specific stoichiometric economics, we fitted observed-variable structural equation models (SEMs). First, we quantified the α-diversity of trees and shrubs. α-diversity was described using species richness (S), the Shannon-Wiener diversity index, Simpson’s diversity index, and Pielou’s evenness index with the R package vegan (Oksanen et al., 2012). These indices were calculated from species-by-plot dominance matrices for each life form. Within each plot, tree dominance was quantified as relative basal area at breast height, whereas shrub dominance was quantified as relative basal area. Species richness was used as the richness metric, Pielou’s index represented evenness, and the Shannon-Wiener and Simpson indices described overall diversity.

Second, potential predictors of PC1 scores were screened by Spearman’s rank correlation. Variables significantly associated with PC1 were first retained at *p* ≤ 0.05, followed by recursive selection of related environmental variables according to the predefined shrub-layer, tree-layer, and other variables. Among highly collinear predictors (|r| > 0.8), only the variable with greater ecological interpretability for bamboo stoichiometric economics was kept for subsequent analysis.

Finally, based on *a priori* ecological hypotheses, we specified a hypothesized path model using the R package lavaan (Rosseel, 2012). In this model, environmental, tree-layer, and shrub-layer variables were specified to have direct paths to bamboo stoichiometric economics. Tree-layer variables were modelled as responses to environmental variables, whereas shrub-layer variables were modelled as responses to both environmental and tree-layer variables. This structure reflects the assumption that tree-layer attributes may regulate the understory microenvironment via canopy effects, while shrub-layer attributes may capture potential competitive effects within the understory. It also allows environmental variables to be associated with bamboo stoichiometric economics both directly and indirectly through tree- and shrub-layer pathways.

All variables were z-score standardized before analysis, and exogenous variables were treated as fixed. Model-level fit was evaluated using the MLR estimator, including the χ^2^ test, degrees of freedom, CFI, TLI, RMSEA, SRMR, AIC, and BIC. Standardized path coefficients (β) were summarized in path diagrams. To quantify uncertainty in direct, indirect, and total effects, we used bootstrap confidence intervals based on 2000 resamples. Explanatory power was summarized as R^2^ for each endogenous variable.

## 3 Results

### 3.1 Stoichiometric trait coordination across shoot and mature stages

**Figure 2:**
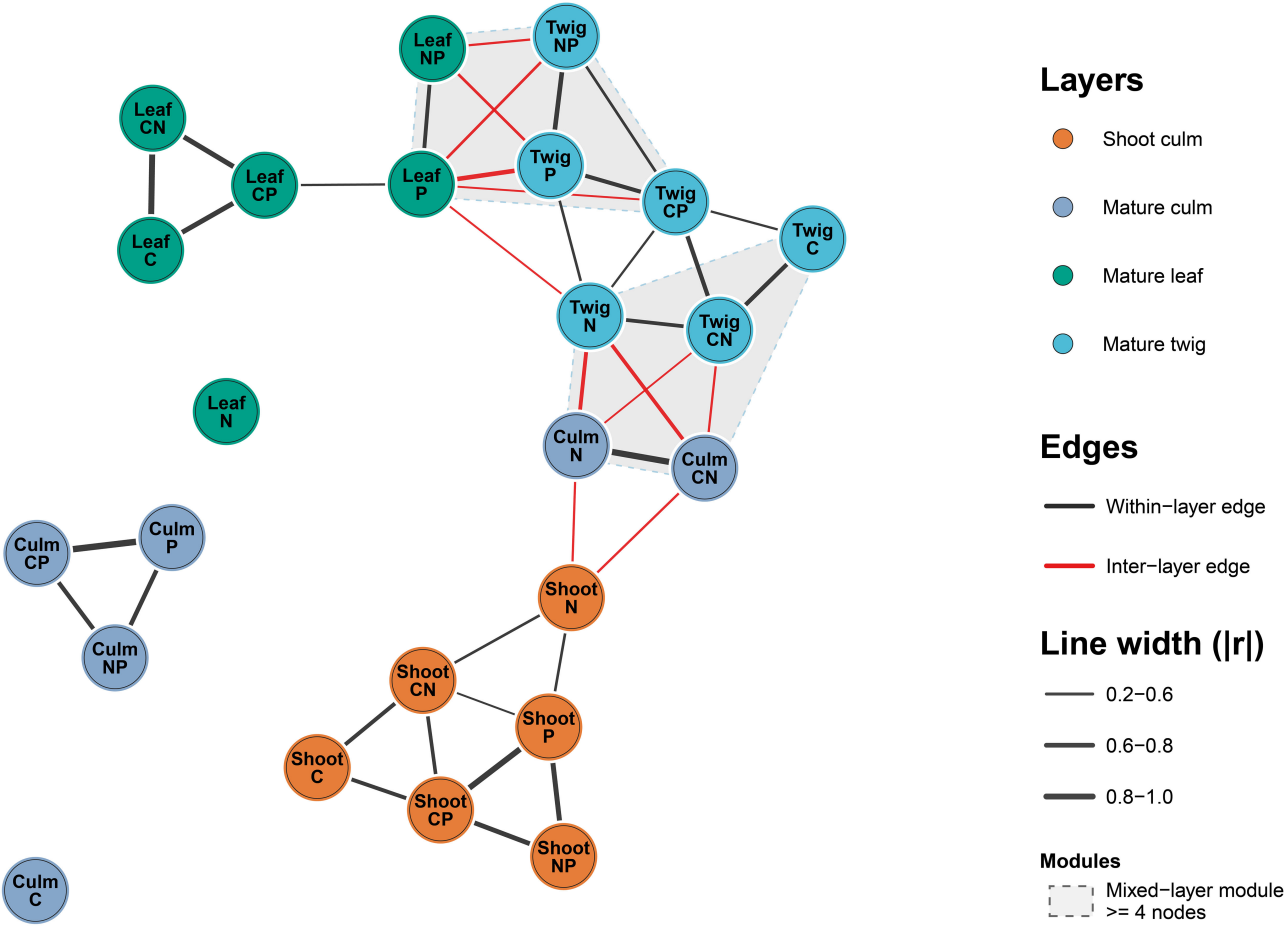
A multilayer network of *Fargesia denudata* stoichiometric traits. Onlycorrelations meeting the retained-edge criteria are shown (|r| > 0.2, FDR-adjusted *p* ≤ 0.05). In the network, Shoot refers to shoot culm, whereas Culm, Leaf, and Twig represent traits only at the mature stage. C, C concentration; N, N concentration; P, P concentration; CN, C:N ratio; CP, C:P ratio; NP, N:P ratio. The complete pairwise correlation table is provided in **Supplementary Table 1**.

Spearman correlation networks showed clear stage- and organ-specific coordination of stoichiometric traits (**Figure 2**). Across all tested independent trait pairs, 12 correlations were retained after FDR correction, including three within-layer and nine interlayer edges.

In shoot culms, one within-layer correlation was retained, with N concentration positively correlated with P concentration (r = 0.531). After maturation, mature twigs retained two within-layer correlations: N concentration was positively correlated with P concentration (r = 0.533) and negatively correlated with C:P ratio (r = −0.487). Mature culms and mature leaves showed no retained within-layer correlations.

Interlayer correlations were sparse but informative. Cross-stage links between shoot and mature culms were retained only for N-related traits, including shoot N–mature culm N (r = 0.485) and shoot N–mature culm C:N (r = −0.495). This indicates that retained cross-stage coordination between shoot and mature culms was mainly N-related.

Within mature bamboo, cross-organ correlations were unevenly distributed. Mature culms were connected to mature twigs mainly through N and C:N traits, including mature culm N–twig N (r = 0.672), and mature culm C:N–twig C:N (r = 0.493). Mature leaves were connected to mature twigs mainly through P and N:P traits, including leaf P–twig P (r = 0.754), leaf P–twig N:P (r = −0.543), leaf N:P–twig P (r = −0.548), and leaf N:P–twig N:P (r = 0.493). No direct retained correlations were detected between mature culm and mature leaf traits.

### 3.2 Stoichiometric-economic axes at organ and aboveground levels

**Figure 3:**
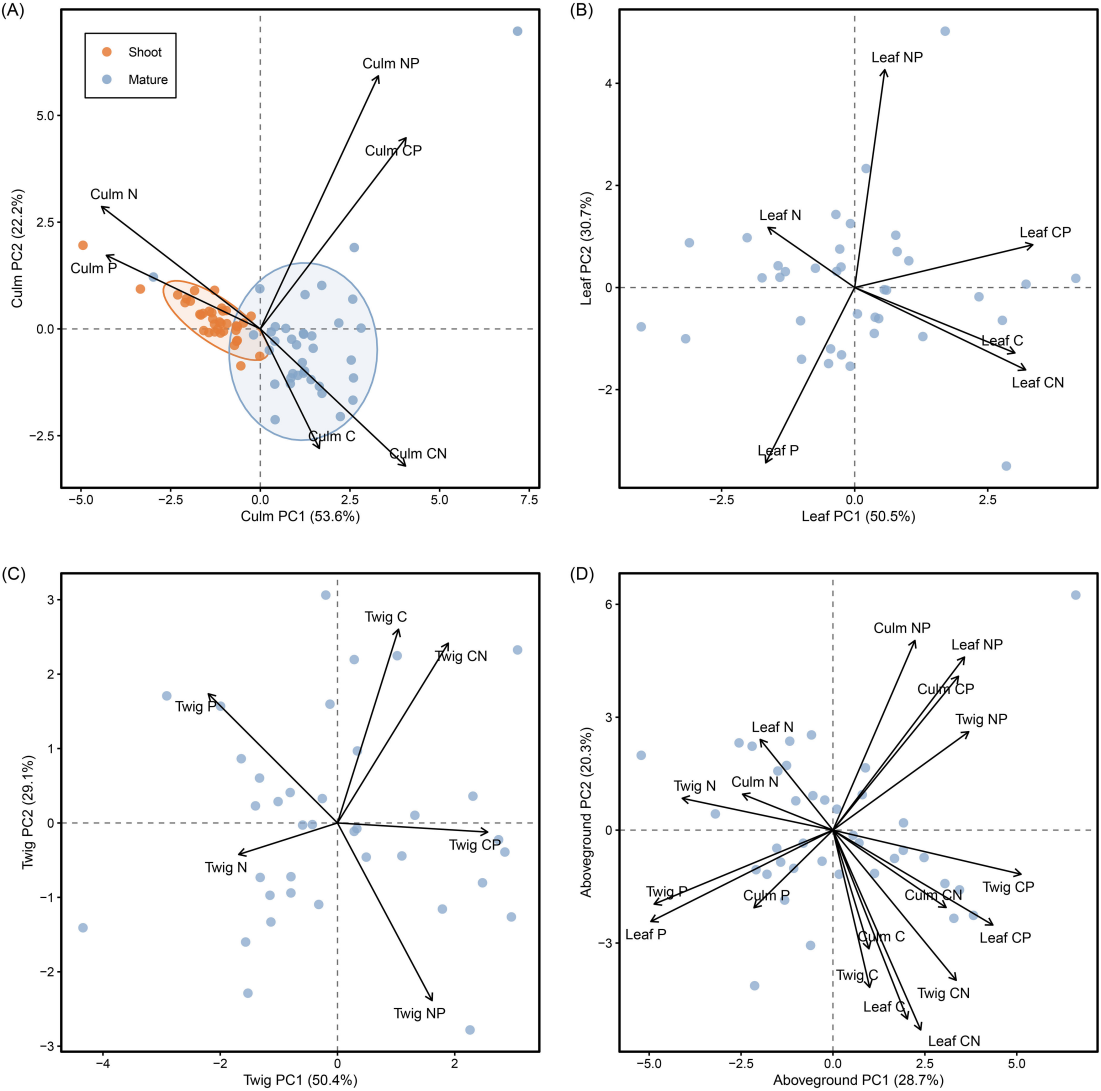
Principal component analysis (PCA) of *Fargesia denudata.* **(A)** Culm PCA of shoot and mature *F. denudata*, **(B)** leaf PCA of mature *F. denudata*, **(C)** twig PCA of mature *F. denudata*, **(D)** aboveground-level PCA of mature *F. denudata*. Arrows indicate trait loadings. Ellipses represent 95% confidence ellipses. C, C concentration; N, N concentration; P, P concentration; CN, C:N ratio; CP, C:P ratio; NP, N:P ratio. Each point represents a plot mean.

PC1 explained the largest proportion of variation in each PCA. For culm traits, PC1 explained 53.6% of the total variation (**Figure 3A**), loaded negatively on N and P concentrations, and positively on C:N, C:P, and N:P ratios, whereas C concentration contributed weakly (**Table 2**). By comparison, shoot culms showed significantly lower PC1 scores than mature culms (Wilcoxon signed-rank test, Z = -6.000, p < 0.001), with median [IQR] values of -1.186 [-1.595, -0.719] and 1.208 [0.710, 1.720], respectively.

**Table 2:** PCA loadings and Spearman’s rank correlation coefficients between stoichiometric traits and PC1 axes.

Traits	Loading (Spearman’s r; significance)			
	Culm PC1	Leaf PC1	Twig PC1	Mature aboveground PC1
Culm C	0.179 (0.358 ^**^)			0.069 (0.127)
Culm N	-0.482 (-0.898 ^**^)			-0.174 (-0.551 ^**^)
Culm P	-0.467 (-0.947 ^**^)			-0.152 (-0.378 ^*^)
Culm CN	0.441 (0.924 ^**^)			0.218 (0.573 ^**^)
Culm CP	0.442 (0.970 ^**^)			0.241 (0.404 ^*^)
Culm NP	0.358 (0.505 ^**^)			0.158 (0.072)
Leaf C		0.499 (0.791 ^**^)		0.143 (0.268)
Leaf N		-0.27 (-0.369 ^*^)		-0.14 (-0.245)
Leaf P		-0.277 (-0.458 ^**^)		-0.35 (-0.757 ^**^)
Leaf CN		0.533 (0.880 ^**^)		0.169 (0.329)
Leaf CP		0.555 (0.967 ^**^)		0.307 (0.610 ^**^)
Leaf NP		0.094 (0.220)		0.253 (0.534 ^**^)
Twig C			0.224 (0.397 ^*^)	0.071 (0.178)
Twig N			-0.363 (-0.683 ^**^)	-0.29 (-0.675 ^**^)
Twig P			-0.475 (-0.772 ^**^)	-0.344 (-0.815 ^**^)
Twig CN			0.408 (0.757 ^**^)	0.237 (0.548 ^**^)
Twig CP			0.552 (0.946 ^**^)	0.362 (0.802 ^**^)
Twig NP			0.348 (0.464 ^**^)	0.261 (0.495 ^**^)

^*^ *p* ≤ 0.05, ^**^ *p* ≤ 0.01. C, C concentration; N, N concentration; P, P concentration; CN, C:N ratio; CP, C:P ratio; NP, N:P ratio.

For mature leaf traits, PC1 explained 50.5% of the total variation (**Figure 3B**) and represented a gradient from higher C concentration, C:N, and C:P ratios to higher N and P concentrations. N:P ratio contributed weakly and was not significantly associated with PC1 (**Table 2**). For mature twig traits, PC1 explained 50.4% of the total variation (**Figure 3C**), with positive contributions from C:N, C:P, and N:P ratios, and a weaker positive contribution from C concentration, whereas P and N concentrations contributed negatively (**Table 2**).

At the mature aboveground level, PC1 explained 28.7% of the total variation (**Figure 3D**). This integrated axis mainly separated higher stoichiometric ratios from higher N and P concentrations. It loaded positively on culm CN, culm CP, leaf CP, leaf NP, twig CN, twig CP, and twig NP, and negatively on culm N, culm P, leaf P, twig N, and twig P. Culm C, culm NP, leaf C, leaf N, leaf CN, and twig C all contributed weakly or nonsignificantly to PC1 (**Table 2**).

### 3.3 Scaling relationships of PC1 scores at organ and aboveground levels

**Figure 4:**
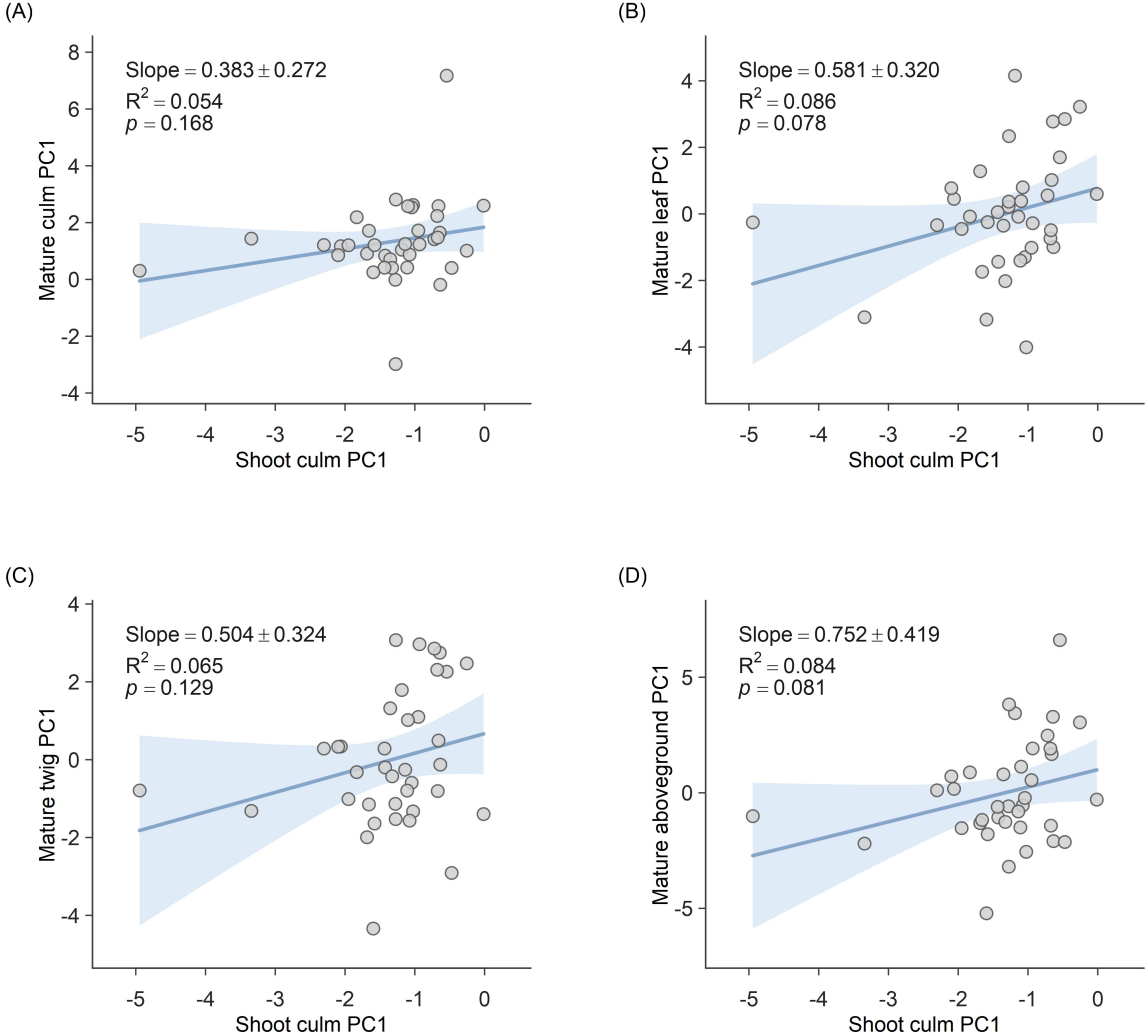
Relationships between shoot-culm PC1 and **(A)** mature culm PC1, **(B)** mature leaf PC1, **(C)** mature twig PC1, and **(D)** mature aboveground PC1. Lines indicate fitted linear regressions, and shaded areas represent 95% confidence intervals. Each point represents a plot mean.

Scaling analyses revealed stage-dependent relationships among PC1 scores. Shoot-culm PC1 showed no statistically detectable relationships with mature culm, leaf, twig, or integrated mature aboveground PC1 scores, although weak trends were observed for some relationships (all *p* > 0.05; **Figures 4A–D**).

**Figure 5:**
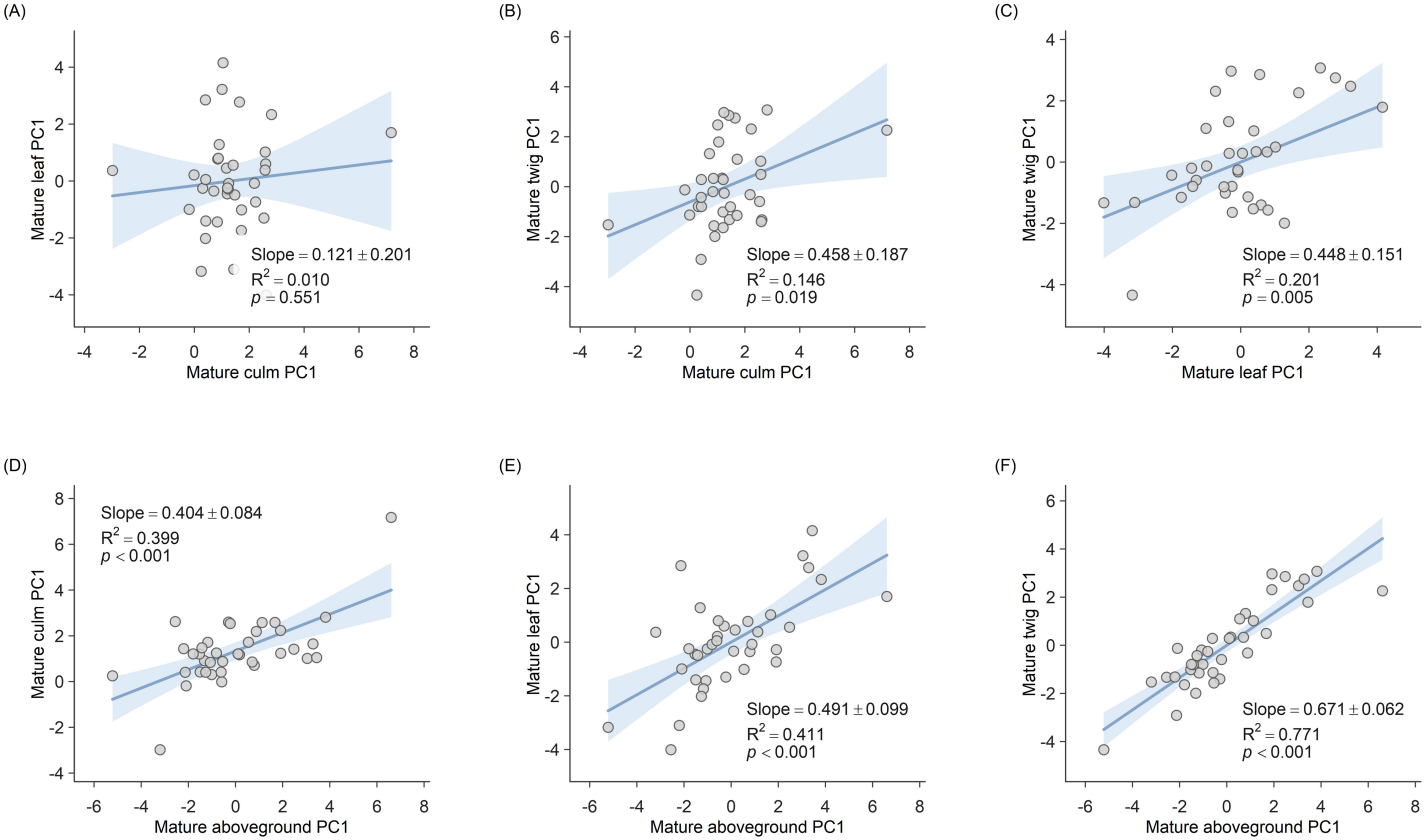
Scaling relationships of organ- and aboveground-level PC1 scores in mature *Fargesia denudata*. **(A–C)** Scaling relationships among culm, leaf, and twig PC1 scores. **(D–F)** Scaling relationships between organ-level PC1 scores and aboveground PC1 scores. Blue lines indicate fitted regression lines, and shaded areas represent 95% confidence intervals. Each point represents a plot mean.

In mature *F. denudata*, culm and leaf PC1 scores were not significantly related (*p* = 0.551; **Figure 5A**), whereas twig PC1 scaled positively with culm PC1 and leaf PC1 (*p* ≤ 0.05; **Figures 5B, C**). Mature aboveground PC1 was positively associated with all organ-level PC1 scores (*p* < 0.001; **Figures 5D–F**). Among these organ-level associations, the strongest relationship was observed between mature aboveground PC1 and twig PC1 (R^2^ = 0.771; **Figure 5F**).

### 3.4 Environmental and community differences between plots with and without *F. denudata*

Independent sample tests showed that 2 of the 18 environmental and community variables differed significantly between plots with and without *F. denudata* (*p* ≤ 0.05) (**Table 3**). Plots with *F. denudata* had steeper slopes and higher soil C concentration than plots without *F. denudata*. In contrast, no significant differences were detected for other environmental or community variables (*p* > 0.05) (**Supplementary Table 2**).

**Table 3:** Environmental and community variables that differed significantly between plots with and without *Fargesia denudata*.

Variables	Median [IQR]		U	*p*
	Plots with *F. denudata* (N = 37)	Plots without *F. denudata* (N = 15)		
Slope (°)	12 [8, 18]	6 [5, 8]	428.5	0.002
Soil C concentration (g/kg)	91.83 [61.52, 138.54]	56.53 [33.78, 92.76]	409	0.008

All comparisons were performed using Mann–Whitney U tests.

### 3.5 Environmental and community associations with PC1 scores

**Figure 6:**
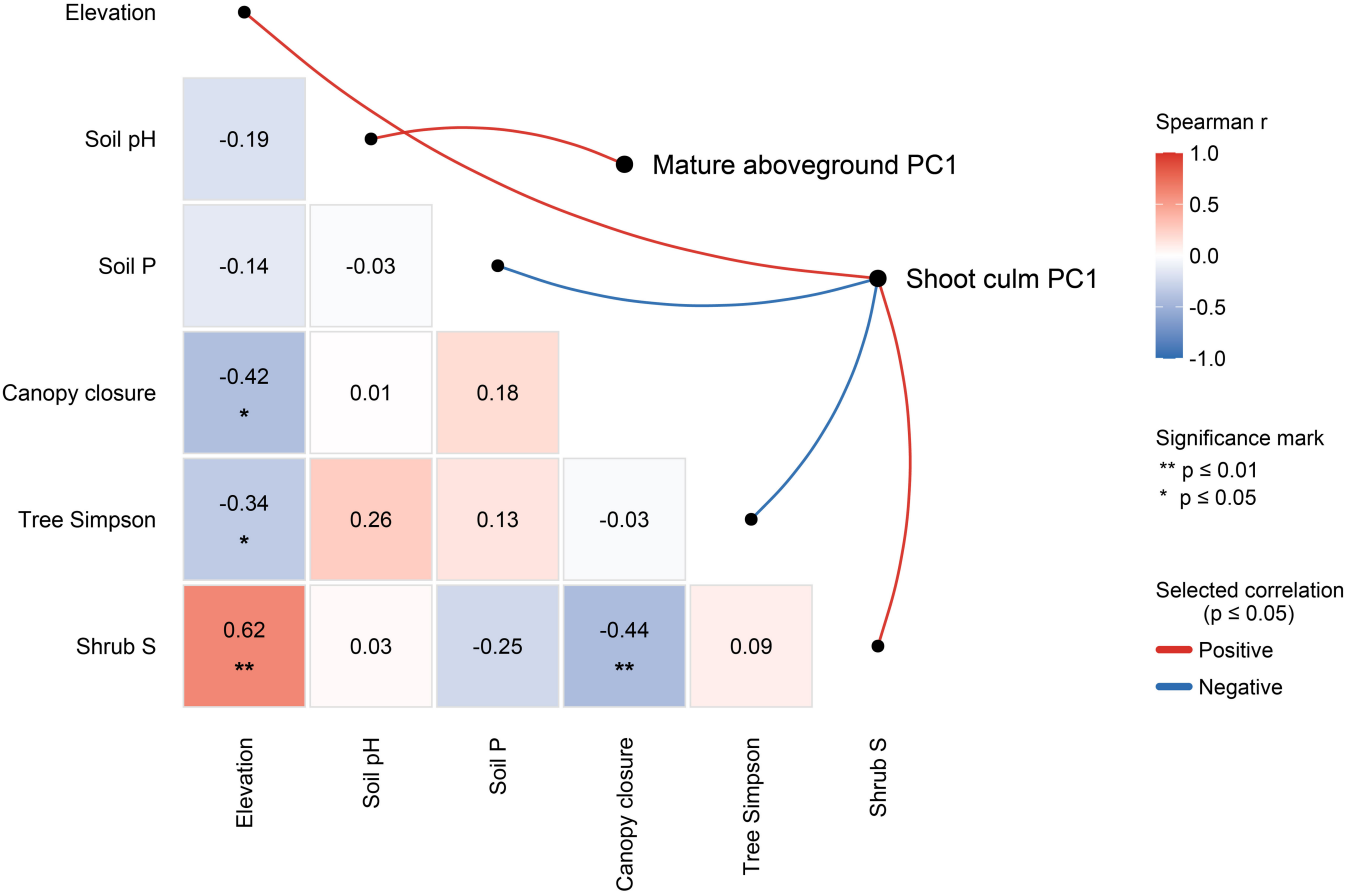
Spearman’s rank correlation screening of environmental and community predictors for PC1 scores. Red and blue arrows indicate positive and negative selected correlations, respectively. ** and * indicate p ≤ 0.01 and p ≤ 0.05.

Correlations between PC1 and environmental and community variables differed between growth stages (**Figure 6**). For shoot culms, PC1 was positively correlated with elevation (r = 0.441, *p* = 0.006) and shrub species richness (r = 0.330, *p* = 0.046), but negatively correlated with soil P (r = -0.362, *p* = 0.028) and tree Simpson diversity (r = -0.340, *p* = 0.039). For mature aboveground PC1, only a single significant bivariate association was detected, with soil pH (r = 0.332, *p* = 0.045), which did not warrant construction of a separate SEM.

**Figure 7:**
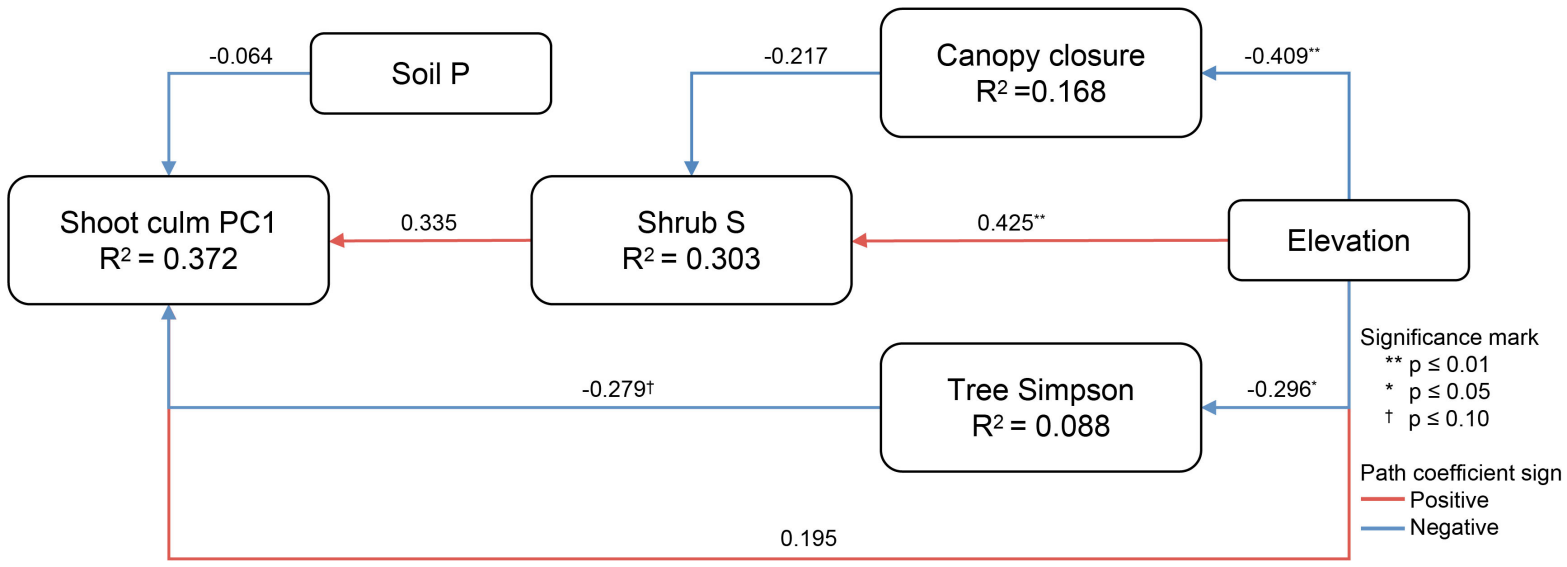
Structural equation model of *Fargesia denudata*. Standardized path coefficients (β) are shown along arrows. R^2^ values are given for endogenous variables. Red and blue arrows indicate positive and negative standardized path coefficients, respectively. **, *, and † indicate p ≤ 0.01, p ≤ 0.05, and p ≤ 0.10, respectively.

The shoot-culm SEM showed a marginal overall fit to the data (χ^2^ = 9.877, df = 6, *p* = 0.130; CFI = 0.889; TLI = 0.742; RMSEA = 0.132; SRMR = 0.088) and explained 37.2% of the variation in shoot-culm PC1 (**Figure 7**; **Supplementary Table 3**). Among the direct predictors of shoot-culm PC1, tree Simpson diversity showed the strongest negative association, although this effect was only marginally supported (β = -0.279, *p* = 0.069). The direct effects of shrub species richness (β = 0.335, *p* = 0.205), elevation (β = 0.195, *p* = 0.123), and soil P (β = -0.064, *p* = 0.710) were not statistically significant.

When direct and mediated pathways were considered jointly, elevation showed a significant positive total effect on shoot-culm PC1 (β = 0.449, *p* = 0.035; **Supplementary Table 4**). However, the specific indirect pathways linking elevation to shoot-culm PC1 through tree Simpson diversity, shrub species richness, or the canopy closure–shrub richness pathway were weak and not statistically supported by bootstrap confidence intervals.

## 4 Discussion

### 4.1 Organ- and stage-specific modularity in aboveground stoichiometric coordination

Within organs and across development, correlations among stoichiometric traits indicate the degree to which organ chemistry is coordinated, and thus where trade-offs are expressed or where traits vary more independently (Kleyer and Minden, 2015; Zhou et al., 2022). Our networks showed that independent within-organ coordination was detected mainly in shoot culms and mature twigs. In shoot culms, the only retained within-layer relationship was the positive correlation between N and P concentrations, suggesting coordinated variation in nutrient status during early culm development. Mature twigs retained a slightly more complex internal structure, with a positive N–P relationship and a negative association between N concentration and the C:P ratio. In contrast, mature culms and mature leaves showed no retained independent within-organ correlations, suggesting weaker internal integration of stoichiometric variation in these organs. Thus, maturation did not produce a uniform decline or increase in stoichiometric coordination across the plant; rather, independent within-organ coordination became concentrated in mature twigs. This pattern is consistent with an allocation-based view of plant traits, in which stoichiometric coordination reflects the contrasting functional roles of different organs rather than a single whole-plant level of integration (Kleyer and Minden, 2015). In this context, the retained coordination in mature twigs may reflect their intermediate role in linking structural support, nutrient transfer, and leaf function.

Cross-stage and cross-organ coordination was sparse, but it showed clear trait-specific organization. The retained links were centered mainly on N- and P-related traits rather than C concentration, consistent with the view that N and P often act as key elements linking functional and stoichiometric variation within and across plant organs (Kleyer and Minden, 2015; Li et al., 2022), consistent with evidence that N and P can covary within and across plant organs (Tian et al., 2018; Liu et al., 2024). Cross-stage links between shoot and mature culms were limited to N-related traits, including the positive association between shoot N and mature culm N and the negative association between shoot N and mature culm C:N. This suggests partial continuity in N status during culm maturation and may be related to clonal integration in bamboo (Zhao and Cai, 2023). Within mature bamboo, culm–twig coordination was mainly associated with N- and C:N-related traits, whereas twig–leaf coordination was mainly associated with P and N:P traits. The absence of direct retained links between mature culms and leaves suggests that mature aboveground stoichiometric coordination was not organized through direct culm–leaf coupling. Instead, mature twigs appeared to form the main topological bridge connecting culms and leaves. This bridge-like structure may reflect organ differentiation and transport architecture (Zhu et al., 2023), although the underlying mechanism requires further testing.

PCA summarized these coordination patterns into dominant PC1 axes of shared stoichiometric variation. Across culm, mature leaf, mature twig, and mature aboveground analyses, PC1 generally separated N- and P-enriched stoichiometry from ratio-enriched stoichiometry. This configuration is consistent with the expected contrast between nutrient-enriched, relatively acquisitive tissues and more conservative, ratio-enriched tissues, mainly reflected by higher C:N and C:P (Wright et al., 2004; Donovan et al., 2011; Reich and Cornelissen, 2014). In a stoichiometric context, this interpretation is also supported by studies linking plant C:N:P traits with nutrient allocation and functional strategies (Tian et al., 2021). Thus, lower PC1 scores generally corresponded to the nutrient-enriched, relatively acquisitive end of the spectrum, whereas higher PC1 scores corresponded to the ratio-enriched, more conservative end.

### 4.2 Ontogenetic reassembly of the aboveground stoichiometric-economic spectrum

Bamboos are clonal plants, and ramets of different sizes and ages within a plot can be connected by rhizomes (Liese and Köhl, 2015). Such connections allow resource sharing and support the use of plot-level stoichiometric patterns as a biologically meaningful scale of analysis (Zhai et al., 2022). However, aboveground stoichiometric economics is expressed through different organ sets across ontogeny. In the shoot stage, aboveground stoichiometry was represented by shoot culms, whereas in the mature stage it reflected the combined contributions of culms, leaves, and twigs (Li et al., 2023b).

Against this developmental backdrop, culm PC1 revealed a clear ontogenetic shift in stoichiometric strategy. Shoot culms had significantly lower PC1 scores than mature culms, placing them toward the nutrient-enriched, relatively acquisitive end of the spectrum. Mature culms were positioned toward the ratio-enriched, more conservative end. This shift is consistent with rapid culm elongation and high nutrient demand during the shoot stage, followed by nutrient dilution and increasing structural investment as culms mature and lignify (Liese and Köhl, 2015; Ouyang et al., 2024).

However, scaling analyses showed no statistically detectable relationships between shoot-culm PC1 and the PC1 scores of mature culms, twigs, leaves, or integrated mature aboveground traits. This result suggests that shoot-culm stoichiometric economics did not continue directly into mature organ-level or mature aboveground economics. This decoupling is consistent with the view that plant functional strategies can shift across ontogeny rather than remain fixed through development (Barton, 2024). In *F. denudata*, shoot-culm PC1 likely reflects a transient nutrient-enriched, relatively acquisitive strategy during early culm development. Mature aboveground PC1, by contrast, reflects the combined stoichiometric contributions of mature culms, leaves, and twigs. The lack of significant scaling relationships therefore suggests that mature stoichiometric economics cannot be inferred from shoot-culm economics alone. This may reflect nutrient dilution, organ differentiation, and changing allocation demands during bamboo culm maturation (Ouyang et al., 2024).

Different organs may contribute unequally to integrated stoichiometric economics (Li et al., 2022). In mature *F. denudata*, organ-level PC1 relationships indicated an organ-specific assembly of aboveground stoichiometric economics. Culm and leaf PC1 scores were not significantly related, suggesting that mature culm and leaf economics were not directly coupled. By contrast, twig PC1 scaled positively with both culm and leaf PC1, indicating that twigs formed the main statistical link between these two organs. Mature aboveground PC1 was positively associated with all organ-level PC1 scores, but its strongest association was with twig PC1. This pattern suggests that mature aboveground stoichiometric economics integrated contributions from culms, leaves, and twigs, while being most closely represented by twig economics. This organ-specific integration is consistent with whole-plant economics frameworks in which organ-level traits are coordinated but not uniformly integrated (Zhao et al., 2017; Li et al., 2022). It also provides a structural parallel to compartmentalized homeostasis, which emphasizes tissue-specific decoupling in nutrient regulation (Wang et al., 2026). Although our study did not directly quantify homeostatic responses to soil nutrient availability, these organ-level relationships suggest that twigs may serve as an intermediate component of mature aboveground stoichiometric integration. Thus, twigs appeared to function as a topological bridge and a key contributor to the mature aboveground stoichiometric-economic spectrum.

### 4.3 Stage-dependent associations with the stoichiometric economics spectrum

The habitat of *F. denudata* is often described broadly as occurring in forest understories between 1920 and 3200 m a.s.l (Li, 2018). Our results show that plots with *F. denudata* differed from plots without *F. denudata* mainly in slope and soil C concentration, with plots with *F. denudata* occurring on steeper terrain and containing higher soil C. Steeper slopes may reflect greater local topographic complexity (Badgley et al., 2017), whereas higher soil C concentration may be linked to greater organic matter accumulation (Paul, 2016). These findings refine the broad understory habitat description of *F. denudata* by identifying slope and soil C concentration as the clearest local correlates of its occurrence.

The stoichiometric-economic spectrum showed stage-dependent associations with environmental and community variables. Significant bivariate correlations were concentrated in shoot culms, with shoot-culm PC1 associated with elevation, shrub species richness, soil P, and tree Simpson diversity. In contrast, mature aboveground PC1 showed only one significant association, with soil pH. This pattern indicates that early-stage culms were more closely linked to environmental and community variation, whereas mature aboveground tissues showed weaker measured associations. Such ontogenetic contrast is consistent with evidence that trait-environment and trait-community relationships can shift across growth stages (Henn and Damschen, 2021; Barton, 2024).

The SEM provided a more detailed, although still cautious, view of the pathways associated with shoot-culm stoichiometric economics. The model explained 37.2% of the variation in shoot-culm PC1, but its marginal overall fit indicates that the specific paths should not be interpreted as strong causal evidence. Within this limitation, the most consistent result was the significant positive total effect of elevation on shoot-culm PC1. Mountain elevational gradients can reorganize both abiotic conditions and plant communities, thereby influencing plant nutrient-use strategies (Körner, 2007; Sundqvist et al., 2013). In this study, elevation showed a significant positive total effect on shoot-culm PC1, but neither its direct pathway nor the specific indirect pathways through tree Simpson diversity, canopy closure, or shrub species richness were statistically supported. Shoot culms tended to shift toward the high-PC1 end of the stoichiometric-economic axis at higher elevations, suggesting a more conservative stoichiometric strategy under high-elevation conditions. The total elevational effect may be associated with lower temperatures, shorter growing seasons, reduced soil temperature and slower nutrient cycling at higher elevations (Mayor et al., 2017; Sundqvist et al., 2013; Körner, 2007), all of which can constrain photosynthesis and plant growth and favor nutrient conservation rather than rapid resource acquisition.

Elevation was also associated with changes in the plant community surrounding *F. denudata*. Overstory structure and co-occurring understory vegetation have been shown to influence the growth and distribution of *F. denudata* (Kang et al., 2019; Zangy et al., 2021; Xiong et al., 2022). With increasing elevation, stronger environmental filtering may reduce tree-layer diversity, while associated changes in stand structure may lead to lower canopy closure, resulting in lower tree Simpson diversity and a more open canopy (Xiong et al., 2022; Bañares-de-Dios et al., 2024). Such reductions in overstory constraint may weaken shading and partially release understory plants from light limitation, thereby shifting shoot-culm PC1 toward the acquisitive end of the stoichiometric-economic axis. The decline in canopy closure may increase understory light availability and resource heterogeneity, thereby facilitating shrub-layer recruitment and expansion and potentially increasing shrub species richness (Barbier et al., 2008). In shrub-rich communities, shoot culms tended to adopt a more competition-tolerant and resource-conservative stoichiometric strategy, probably because intensified lateral competition for light, nutrients, and space imposed additional constraints on growth. However, among these modeled community-related pathways, only the path from tree Simpson diversity to shoot-culm PC1 was marginally supported at the 0.10 level, whereas the effects of canopy closure and shrub species richness were not significant.

Overall, although the reduction in overstory diversity and canopy closure at higher elevations may partially promote resource acquisition by releasing *F. denudata* from canopy limitation, this positive community effect appeared insufficient to offset the stronger abiotic constraints and increased shrub-layer competition associated with elevation. Therefore, the overall association with increasing elevation was a shift of shoot culms toward a more conservative stoichiometric strategy along the stoichiometric-economic axis.

## 5 Conclusions

This study shows that stoichiometric economics in *Fargesia denudata* is coordinated but strongly organ- and stage-specific. Stoichiometric traits formed recognizable stoichiometric-economic axes, but their coordination differed among organs. Independent within-organ coordination was limited and was retained mainly in shoot culms and mature twigs, particularly in mature twigs. Across ontogeny, shoot-culm economics did not scale significantly into mature aboveground economics. Instead, mature aboveground stoichiometric economics was assembled through organ-specific contributions, with twigs forming the main link between mature culms and leaves. Environmental and community associations were also stage-dependent, being stronger and more numerous in shoot culms than in mature aboveground tissues. In shoot culms, elevation showed a significant positive total effect on PC1, while tree Simpson diversity showed a marginal negative direct association. Mature aboveground PC1 showed only a limited association with soil pH. Together, these results indicate that *F. denudata* stoichiometric strategies are shaped by organ-specific coordination, ontogenetic reorganization, and stage-dependent links with environmental and community variables.

## Data Availability

The original contributions presented in the study are included in the article/**Supplementary Material**. Further inquiries can be directed to the corresponding author.

## Supplementary material

Supplementary Table 1 Pairwise Spearman’s rank correlation among bamboo stoichiometric traits;

Supplementary Table 2 Differences in environmental and community variables between plots with and without *Fargesia denudata*;

Supplementary Table 3 Structural equation model for shoot-culm PC1;

Supplementary Table 4 Total and indirect effects related to shoot-culm PC1 in the structural equation model.
