# Supplementary material for: Organ-specific and stage-dependent aboveground stoichiometric economics in Fargesia denudata
Source: Front Plant Sci. 2026 Jul 21;17:1882972. doi: 10.3389/fpls.2026.1882972 (PMC13433447; doi:10.3389/fpls.2026.1882972)
Supplement: Supplementary file 1 [file Table1.pdf]

Supplementary materials

SUPPLEMENTARY TABLE 1 Pairwise Spearman's rank correlation among *Fargesia denudata* stoichiometric traits.

SUPPLEMENTARY TABLE 2 Differences in environmental and community variables between plots with and without *Fargesia denudata*.

SUPPLEMENTARY TABLE 3 Structural equation model results for shoot-culm PC1.

SUPPLEMENTARY TABLE 4 Total and indirect effects related to shoot-culm PC1 in the structural equation model.

SUPPLEMENTARY TABLE 1 Pairwise Spearman's rank correlation among *Fargesia denudata* stoichiometric traits.

Trait 1	Trait 2	r	*p*	FDR *p*	Trait 1	Trait 2	r	*p*	FDR *p*
Shoot C	Shoot N	-0.035	0.838	0.906	Culm C	Leaf CN	0.363	0.027	0.225
Shoot C	Shoot P	-0.328	0.047	0.282	Culm C	Leaf CP	0.255	0.128	0.439
Shoot C	Shoot NP	0.374	0.023	0.212	Culm C	Leaf NP	-0.100	0.558	0.739
Shoot C	Culm C	0.359	0.029	0.228	Culm C	Twig C	0.409	0.012	0.159
Shoot C	Culm N	-0.141	0.404	0.654	Culm C	Twig N	-0.070	0.679	0.850
Shoot C	Culm P	0.053	0.756	0.889	Culm C	Twig P	0.143	0.399	0.654
Shoot C	Culm CN	0.213	0.205	0.527	Culm C	Twig CN	0.243	0.147	0.458
Shoot C	Culm CP	0.010	0.951	0.959	Culm C	Twig CP	0.137	0.419	0.654
Shoot C	Culm NP	-0.109	0.521	0.717	Culm C	Twig NP	-0.113	0.507	0.716
Shoot C	Leaf C	0.351	0.033	0.249	Culm N	Culm P	0.287	0.085	0.387
Shoot C	Leaf N	0.141	0.404	0.654	Culm N	Culm CP	-0.297	0.075	0.349
Shoot C	Leaf P	0.108	0.525	0.717	Culm N	Leaf C	-0.160	0.345	0.634
Shoot C	Leaf CN	0.197	0.242	0.557	Culm N	Leaf N	0.172	0.308	0.622
Shoot C	Leaf CP	0.242	0.149	0.458	Culm N	Leaf P	0.270	0.107	0.429
Shoot C	Leaf NP	-0.033	0.845	0.906	Culm N	Leaf CN	-0.151	0.372	0.647
Shoot C	Twig C	0.299	0.072	0.349	Culm N	Leaf CP	-0.255	0.128	0.439
Shoot C	Twig N	0.048	0.779	0.906	Culm N	Leaf NP	-0.142	0.403	0.654
Shoot C	Twig P	0.113	0.507	0.716	Culm N	Twig C	-0.027	0.874	0.915
Shoot C	Twig CN	0.125	0.461	0.680	Culm N	Twig N	0.672	<0.001	0.001
Shoot C	Twig CP	0.183	0.279	0.587	Culm N	Twig P	0.284	0.088	0.392
Shoot C	Twig NP	0.116	0.494	0.708	Culm N	Twig CN	-0.444	0.006	0.089
Shoot N	Shoot P	0.531	0.001	0.025	Culm N	Twig CP	-0.332	0.044	0.274
Shoot N	Shoot CP	-0.392	0.016	0.197	Culm N	Twig NP	0.091	0.593	0.765
Shoot N	Culm C	-0.013	0.939	0.955	Culm P	Culm CN	-0.320	0.054	0.300
Shoot N	Culm N	0.485	0.002	0.047	Culm P	Leaf C	0.212	0.208	0.527
Shoot N	Culm P	0.373	0.023	0.212	Culm P	Leaf N	0.159	0.346	0.634
Shoot N	Culm CN	-0.495	0.002	0.047	Culm P	Leaf P	0.245	0.144	0.456
Shoot N	Culm CP	-0.358	0.029	0.228	Culm P	Leaf CN	0.150	0.375	0.647
Shoot N	Culm NP	0.009	0.956	0.960	Culm P	Leaf CP	0.068	0.691	0.851
Shoot N	Leaf C	-0.116	0.495	0.708	Culm P	Leaf NP	-0.125	0.462	0.680
Shoot N	Leaf N	0.230	0.170	0.499	Culm P	Twig C	-0.025	0.885	0.916
Shoot N	Leaf P	0.184	0.276	0.587	Culm P	Twig N	0.210	0.212	0.527
Shoot N	Leaf CN	-0.175	0.301	0.622	Culm P	Twig P	0.259	0.121	0.439
Shoot N	Leaf CP	-0.171	0.312	0.622	Culm P	Twig CN	-0.026	0.877	0.915
Shoot N	Leaf NP	0.037	0.827	0.906	Culm P	Twig CP	-0.240	0.152	0.461
Shoot N	Twig C	-0.047	0.783	0.906	Culm P	Twig NP	-0.107	0.529	0.717
Shoot N	Twig N	0.347	0.035	0.249	Culm CN	Leaf C	0.251	0.134	0.440
Shoot N	Twig P	0.302	0.069	0.345	Culm CN	Leaf N	-0.110	0.516	0.717
Shoot N	Twig CN	-0.246	0.142	0.456	Culm CN	Leaf P	-0.193	0.253	0.557
Shoot N	Twig CP	-0.335	0.042	0.274	Culm CN	Leaf CN	0.224	0.182	0.501
Shoot N	Twig NP	-0.107	0.528	0.717	Culm CN	Leaf CP	0.312	0.060	0.322
Shoot P	Shoot CN	-0.457	0.005	0.076	Culm CN	Leaf NP	0.127	0.454	0.676
Shoot P	Culm C	-0.047	0.783	0.906	Culm CN	Twig C	0.139	0.413	0.654
Shoot P	Culm N	0.133	0.433	0.666	Culm CN	Twig N	-0.652	<0.001	0.001
Shoot P	Culm P	0.042	0.806	0.906	Culm CN	Twig P	-0.268	0.109	0.429

Trait 1	Trait 2	r	*p*	FDR *p*	Trait 1	Trait 2	r	*p*	FDR *p*
Shoot P	Culm CN	-0.166	0.326	0.634	Culm CN	Twig CN	0.493	0.002	0.047
Shoot P	Culm CP	-0.036	0.832	0.906	Culm CN	Twig CP	0.386	0.018	0.209
Shoot P	Culm NP	0.159	0.346	0.634	Culm CN	Twig NP	-0.073	0.670	0.850
Shoot P	Leaf C	-0.210	0.212	0.527	Culm CP	Leaf C	-0.096	0.571	0.751
Shoot P	Leaf N	0.149	0.380	0.647	Culm CP	Leaf N	-0.179	0.290	0.606
Shoot P	Leaf P	-0.068	0.688	0.851	Culm CP	Leaf P	-0.222	0.187	0.501
Shoot P	Leaf CN	-0.226	0.178	0.501	Culm CP	Leaf CN	-0.029	0.864	0.914
Shoot P	Leaf CP	-0.120	0.478	0.691	Culm CP	Leaf CP	0.028	0.867	0.914
Shoot P	Leaf NP	0.165	0.328	0.634	Culm CP	Leaf NP	0.095	0.578	0.753
Shoot P	Twig C	-0.154	0.362	0.647	Culm CP	Twig C	0.053	0.755	0.889
Shoot P	Twig N	0.030	0.861	0.914	Culm CP	Twig N	-0.232	0.166	0.493
Shoot P	Twig P	0.129	0.446	0.669	Culm CP	Twig P	-0.252	0.133	0.440
Shoot P	Twig CN	-0.137	0.419	0.654	Culm CP	Twig CN	0.046	0.785	0.906
Shoot P	Twig CP	-0.207	0.220	0.527	Culm CP	Twig CP	0.245	0.143	0.456
Shoot P	Twig NP	-0.261	0.118	0.439	Culm CP	Twig NP	0.101	0.553	0.737
Shoot CN	Culm C	0.271	0.105	0.429	Culm NP	Leaf C	-0.265	0.113	0.437
Shoot CN	Culm N	-0.208	0.217	0.527	Culm NP	Leaf N	-0.042	0.805	0.906
Shoot CN	Culm P	-0.163	0.336	0.634	Culm NP	Leaf P	-0.195	0.248	0.557
Shoot CN	Culm CN	0.261	0.119	0.439	Culm NP	Leaf CN	-0.211	0.211	0.527
Shoot CN	Culm CP	0.221	0.188	0.501	Culm NP	Leaf CP	-0.111	0.511	0.717
Shoot CN	Culm NP	0.034	0.843	0.906	Culm NP	Leaf NP	0.130	0.442	0.667
Shoot CN	Leaf C	0.379	0.021	0.212	Culm NP	Twig C	-0.043	0.801	0.906
Shoot CN	Leaf N	-0.224	0.182	0.501	Culm NP	Twig N	0.185	0.274	0.587
Shoot CN	Leaf P	-0.108	0.524	0.717	Culm NP	Twig P	-0.096	0.573	0.751
Shoot CN	Leaf CN	0.373	0.023	0.212	Culm NP	Twig CN	-0.297	0.074	0.349
Shoot CN	Leaf CP	0.371	0.024	0.212	Culm NP	Twig CP	-0.018	0.913	0.937
Shoot CN	Leaf NP	-0.122	0.472	0.690	Culm NP	Twig NP	0.140	0.410	0.654
Shoot CN	Twig C	0.304	0.067	0.344	Leaf C	Leaf N	-0.056	0.741	0.889
Shoot CN	Twig N	-0.035	0.837	0.906	Leaf C	Leaf P	0.036	0.834	0.906
Shoot CN	Twig P	-0.104	0.542	0.730	Leaf C	Leaf NP	-0.062	0.714	0.870
Shoot CN	Twig CN	0.191	0.257	0.561	Leaf C	Twig C	0.325	0.049	0.282
Shoot CN	Twig CP	0.376	0.022	0.212	Leaf C	Twig N	-0.073	0.668	0.850
Shoot CN	Twig NP	0.272	0.103	0.429	Leaf C	Twig P	-0.039	0.820	0.906
Shoot CP	Culm C	0.144	0.395	0.654	Leaf C	Twig CN	0.278	0.096	0.420
Shoot CP	Culm N	-0.152	0.369	0.647	Leaf C	Twig CP	0.207	0.218	0.527
Shoot CP	Culm P	-0.010	0.951	0.959	Leaf C	Twig NP	0.025	0.882	0.916
Shoot CP	Culm CN	0.203	0.227	0.540	Leaf N	Leaf P	0.367	0.026	0.219
Shoot CP	Culm CP	0.028	0.868	0.914	Leaf N	Leaf CP	-0.211	0.209	0.527
Shoot CP	Culm NP	-0.154	0.362	0.647	Leaf N	Twig C	0.021	0.902	0.930
Shoot CP	Leaf C	0.326	0.049	0.282	Leaf N	Twig N	0.342	0.038	0.261
Shoot CP	Leaf N	-0.057	0.736	0.888	Leaf N	Twig P	0.226	0.179	0.501
Shoot CP	Leaf P	0.014	0.936	0.955	Leaf N	Twig CN	-0.201	0.233	0.548
Shoot CP	Leaf CN	0.270	0.106	0.429	Leaf N	Twig CP	-0.150	0.375	0.647
Shoot CP	Leaf CP	0.257	0.125	0.439	Leaf N	Twig NP	-0.040	0.814	0.906
Shoot CP	Leaf NP	-0.078	0.648	0.832	Leaf P	Leaf CN	-0.092	0.587	0.762
Shoot CP	Twig C	0.258	0.124	0.439	Leaf P	Twig C	0.149	0.377	0.647
Shoot CP	Twig N	-0.037	0.830	0.906	Leaf P	Twig N	0.454	0.005	0.076
Shoot CP	Twig P	-0.136	0.423	0.655	Leaf P	Twig P	0.754	<0.001	<0.001
Shoot CP	Twig CN	0.208	0.216	0.527	Leaf P	Twig CN	-0.153	0.366	0.647

Trait 1	Trait 2	r	*p*	FDR *p*	Trait 1	Trait 2	r	*p*	FDR *p*
Shoot CP	Twig CP	0.334	0.044	0.274	Leaf P	Twig CP	-0.456	0.005	0.076
Shoot CP	Twig NP	0.332	0.045	0.274	Leaf P	Twig NP	-0.543	0.001	0.024
Shoot NP	Culm C	-0.009	0.960	0.960	Leaf CN	Twig C	0.258	0.123	0.439
Shoot NP	Culm N	0.142	0.402	0.654	Leaf CN	Twig N	-0.171	0.310	0.622
Shoot NP	Culm P	0.154	0.363	0.647	Leaf CN	Twig P	-0.138	0.417	0.654
Shoot NP	Culm CN	-0.131	0.439	0.667	Leaf CN	Twig CN	0.296	0.076	0.349
Shoot NP	Culm CP	-0.162	0.338	0.634	Leaf CN	Twig CP	0.229	0.173	0.501
Shoot NP	Culm NP	-0.131	0.439	0.667	Leaf CN	Twig NP	0.070	0.682	0.850
Shoot NP	Leaf C	0.194	0.251	0.557	Leaf CP	Twig C	0.101	0.552	0.737
Shoot NP	Leaf N	-0.054	0.751	0.889	Leaf CP	Twig N	-0.307	0.065	0.339
Shoot NP	Leaf P	0.144	0.395	0.654	Leaf CP	Twig P	-0.426	0.009	0.120
Shoot NP	Leaf CN	0.160	0.345	0.634	Leaf CP	Twig CN	0.221	0.188	0.501
Shoot NP	Leaf CP	0.071	0.677	0.850	Leaf CP	Twig CP	0.349	0.034	0.249
Shoot NP	Leaf NP	-0.174	0.304	0.622	Leaf CP	Twig NP	0.318	0.055	0.300
Shoot NP	Twig C	0.195	0.247	0.557	Leaf NP	Twig C	-0.170	0.313	0.622
Shoot NP	Twig N	0.165	0.330	0.634	Leaf NP	Twig N	-0.185	0.273	0.587
Shoot NP	Twig P	0.060	0.725	0.879	Leaf NP	Twig P	-0.548	<0.001	0.024
Shoot NP	Twig CN	0.035	0.839	0.906	Leaf NP	Twig CN	-0.054	0.752	0.889
Shoot NP	Twig CP	0.036	0.831	0.906	Leaf NP	Twig CP	0.268	0.109	0.429
Shoot NP	Twig NP	0.195	0.248	0.557	Leaf NP	Twig NP	0.493	0.002	0.047
Culm C	Culm N	-0.121	0.477	0.691	Twig C	Twig N	-0.034	0.839	0.906
Culm C	Culm P	-0.196	0.245	0.557	Twig C	Twig P	0.160	0.344	0.634
Culm C	Culm NP	0.065	0.704	0.861	Twig C	Twig NP	-0.139	0.412	0.654
Culm C	Leaf C	0.392	0.016	0.197	Twig N	Twig P	0.533	0.001	0.025
Culm C	Leaf N	0.069	0.684	0.850	Twig N	Twig CP	-0.487	0.002	0.047
Culm C	Leaf P	0.234	0.164	0.493	Twig P	Twig CN	-0.254	0.130	0.439

Shoot refers to shoot culm, whereas culm, leaf, and twig represent traits only at the mature stage.

SUPPLEMENTARY TABLE 2 Differences in environmental and community variables between plots with and without *Fargesia denudata*.

Variables	Mean ± SD / Median [IQR]		Statistic	*p*
	Plots with *F. denudata* (N=37)	Plots without *F. denudata* (N=15)		
Aspect [U]	-0.24 [-0.73, 0.34]	-0.09 [-0.68, 0.13]	245	0.518
Slope (°) [U]	12 [8, 18]	6 [5, 8]	428.5	0.002
Elevation (m) [U]	2811 [2630, 2903]	2655 [2487, 2986]	312	0.492
Soil pH [t]	6.11 ± 0.58	6.44 ± 0.57	-1.828	0.073
Soil EC (μS/cm) [U]	223.00 [155.00, 363.00]	220.00 [187.50, 371.00]	256	0.671
Soil C (g/kg) [U]	91.83 [61.52, 138.54]	56.53 [33.78, 92.76]	409	0.008
Soil N (mg/g) [U]	10.64 [7.29, 15.67]	6.01 [3.90, 10.95]	354	0.125
Soil P (mg/g) [t]	0.85 ± 0.37	0.76 ± 0.42	0.720	0.475
Canopy closure [U]	0.55 [0.40, 0.65]	0.45 [0.38, 0.70]	268.5	0.863
Tree S [U]	5 [4, 7]	6 [4, 6]	287	0.854
Tree Shannon [t]	0.76 ± 0.42	0.73 ± 0.32	0.249	0.804
Tree Simpson [t]	0.40 ± 0.20	0.38 ± 0.18	0.201	0.842
Tree Pielou [t]	0.461 ± 0.222	0.478 ± 0.236	-0.246	0.806
Shrub coverage (%) [U]	30 [20, 55]	40 [15, 65]	261	0.746
Shrub S [U]	6 [5, 6]	4 [2, 7]	355.5	0.113
Shrub Shannon [U]	1.38 [1.06, 1.59]	0.84 [0.17, 1.51]	352	0.135
Shrub Simpson [U]	0.71 [0.53, 0.76]	0.46 [0.08, 0.74]	345	0.175
Shrub Pielou [U]	0.797 [0.659, 0.863]	0.779 [0.120, 0.886]	302	0.627

[t]: Student's t-test, [U]: Mann-Whitney U test. Values are mean ± SD for Student's t-tests and median [IQR] for Mann-Whitney U tests.

SUPPLEMENTARY TABLE 3 Direct effects in the **s**tructural equation model for shoot-culm PC1.

Response	Predictor	Estimate	Std. β	SE	z	*p*	Bootstrap 95% CI
Shoot-culm PC1	Elevation	0.199	0.195	0.129	1.541	0.123	[-0.105, 0.556]
	Soil P	-0.065	-0.064	0.175	-0.372	0.710	[-0.405, 0.291]
	Tree Simpson	-0.285	-0.279	0.157	-1.821	0.069	[-0.632, 0.029]
	Shrub S	0.342	0.335	0.270	1.268	0.205	[-0.155, 0.902]
Shrub S	Canopy closure	-0.217	-0.217	0.136	-1.596	0.111	[-0.516, 0.094]
	Elevation	0.425	0.425	0.120	3.535	<0.001	[0.180, 0.707]
Canopy closure	Elevation	-0.409	-0.409	0.153	-2.676	0.007	[-0.736, -0.089]
Tree Simpson	Elevation	-0.296	-0.296	0.137	-2.158	0.031	[-0.607, 0.010]

SUPPLEMENTARY TABLE 4 Total and indirect effects related to shoot-culm PC1 in the structural equation model

Effect	Estimate	Std. effect	Bootstrap 95% CI	*p*
Canopy closure to shoot-culm PC1 via Shrub S	-0.074	-0.073	[-0.246, 0.074]	0.336
Elevation to shoot-culm PC1 (Total)	0.459	0.449	[0.075, 0.901]	0.035
via Tree Simpson	0.084	0.083	[-0.014, 0.269]	0.246
via Shrub S	0.145	0.142	[-0.056, 0.486]	0.306
via Canopy closure + Shrub S	0.030	0.030	[-0.035, 0.103]	0.350
via Tree Simpson + Canopy closure + Shrub S	0.260	0.255	[-0.073, 0.745]	0.209
